# Supplementary material for: Mobile Host mRNAs Are Translated to Protein in the Associated Parasitic Plant Cuscuta campestris
Source: Plants (Basel). 2021 Dec 28;11(1):93. doi: 10.3390/plants11010093 (PMC8747733; doi:10.3390/plants11010093)
Supplement: Supplementary file 1 [file plants-11-00093-s001.zip › plants-1490272-sm/20211103_Supp_Figs1-3.pdf]

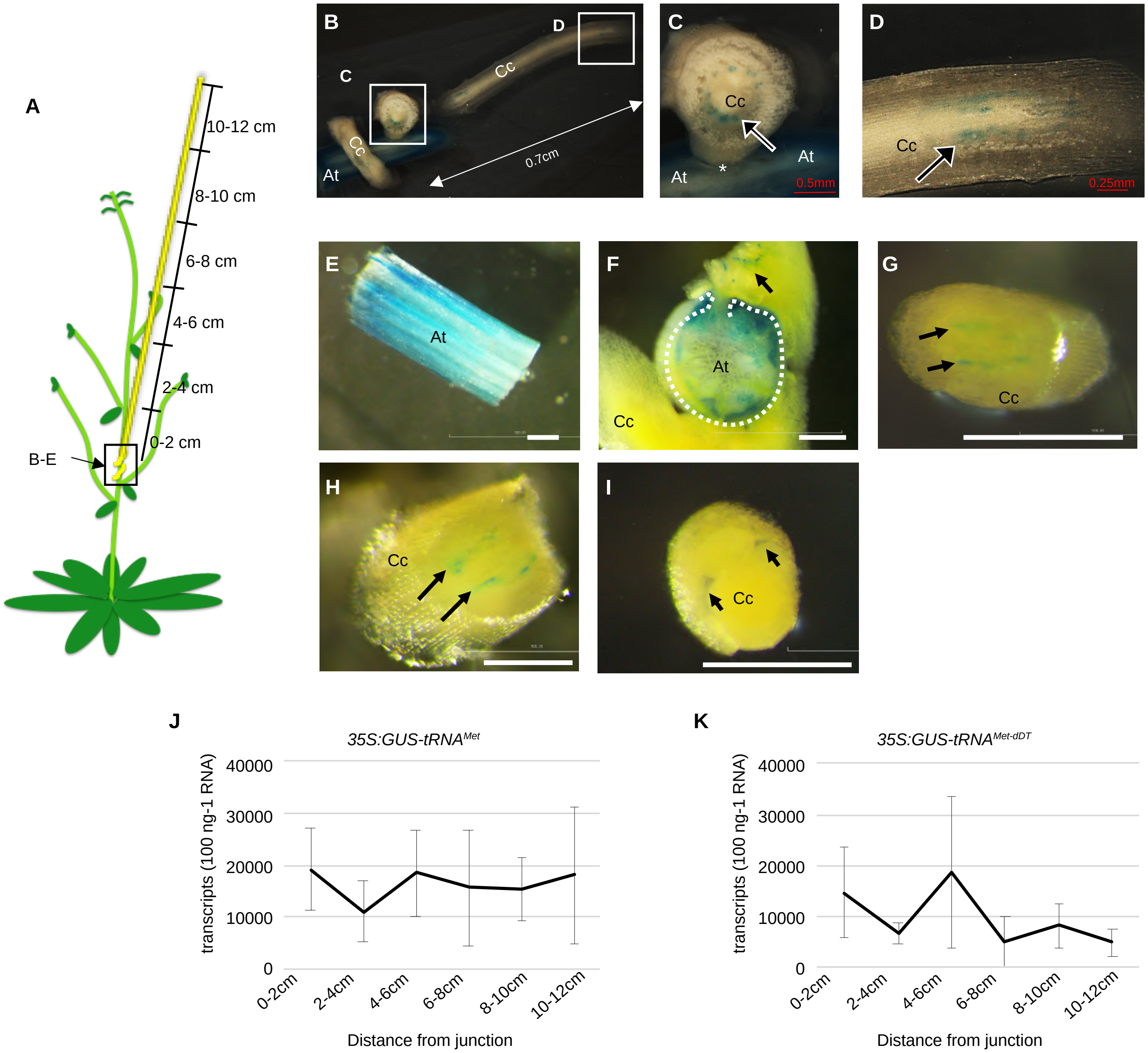

Supplementary Figure 1. GUS staining and quantification of *GUS* transcript levels using *Cuscuta* on *35S:GUS-tRNA*^{Met} and *35S:GUS-tRNA*$^{Met\text{-}dDT}$ *Arabidopsis*. Tissues were separately harvested from different regions (**A**). GUS staining of *Cuscuta* on *35S:GUS-tRNA*^{Met} *Arabidopsis* showed GUS in vascular bundles, several mm from the haustorium (**B**). *Cuscuta* and host plant tissue were longitudinally sectioned, stained and de-stained. High magnification images of B (**C** and **D**). Host stem (**E**), haustorium (**F**), 4-6 cm from junction (**G**), 8-10 cm from junction (**H**), and 10-12 cm from junction (**I**) were stained by X-gluc solution for 72 hours. Black arrows indicate GUS stained regions. White scale bars, 500 µm. Quantification of *GUS* transcripts were performed by real-time RT-PCR (**J** and **K**). Vertical axes are copy numbers of GUS transcript per 100 ng total RNA. Values indicate means and SEs of 3 replicates. No significant differentiation was detected between each location by Tukey–Kramer honestly significant difference (HSD) test ($\alpha < 0.05$).

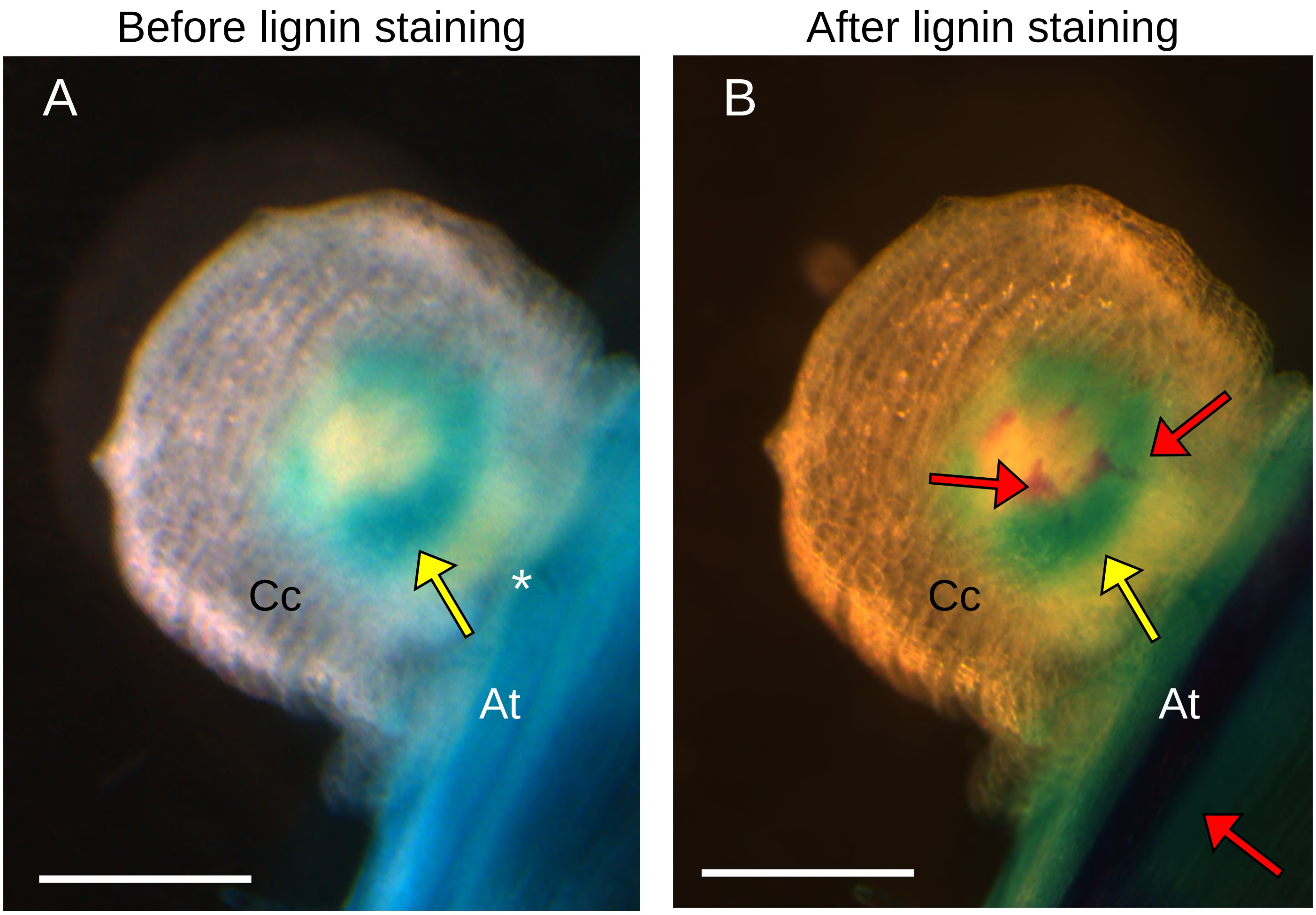

Supplementary Figure 2. Histochemical localization of β-glucuronidase and xylem. *Cuscuta* was inoculated on stems of 3 weeks old *Arabidopsis* (*35S:GUS-tRNA^{Met}*) for a week. Haustoria between *Arabidopsis* and *Cuscuta* were longitudinally cross-sectioned. For the GUS staining, sectioned samples were stained with X-gluc solution for 2 hours (**A**). Without de-staining, same tissue was stained by phloroglucinol-HCl for lignin staining assay, indicated by red arrows (**B**). The blue color of GUS activity in *Cuscuta* is indicated by yellow arrows. Asterisk indicates haustoria. Red arrows indicate the presence of lignin. Scale bar: 500 µm.

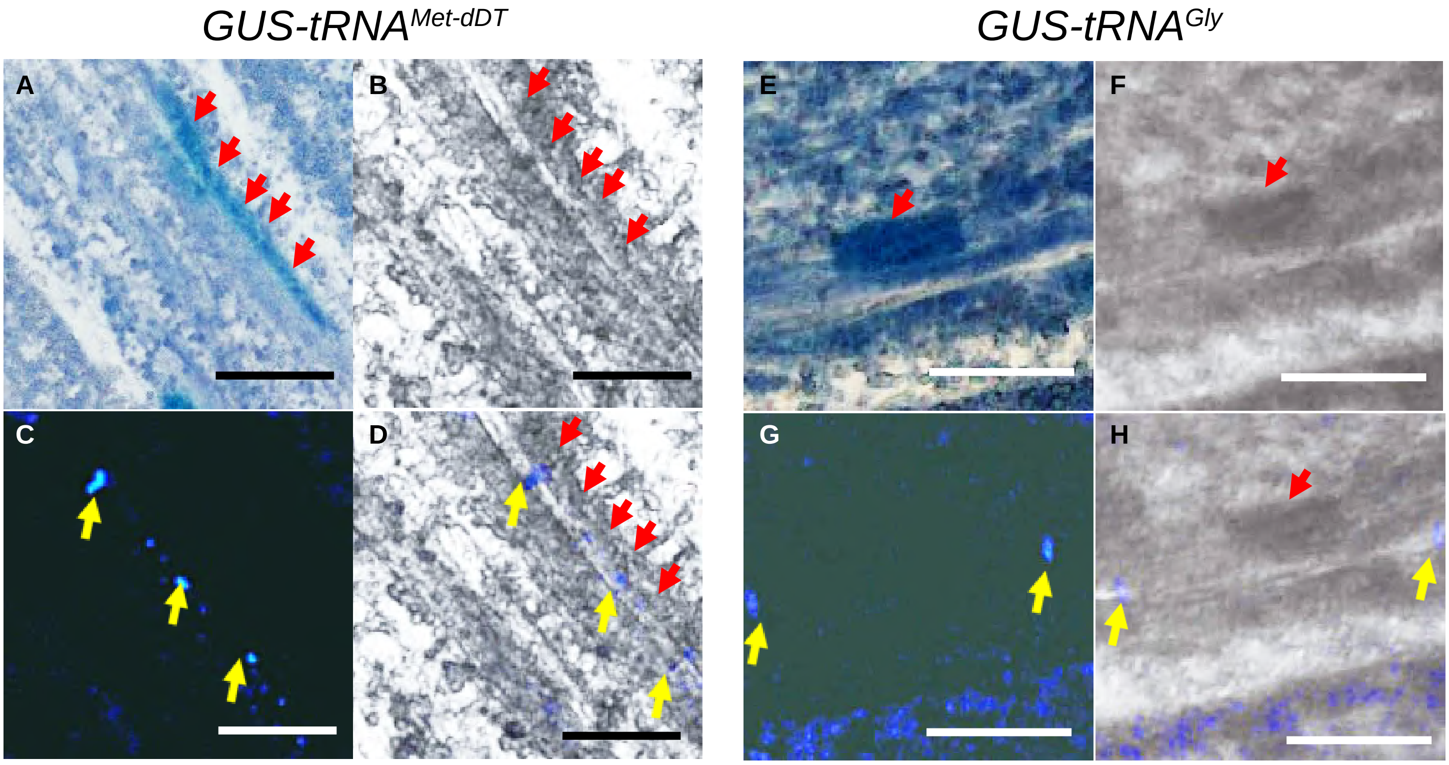

Supplementary Figure 3. GUS activity detected in the cells adjacent to aniline-blue-stained sieve tube. A 20 μm-thickness paraffin section of *Cuscuta* stem growing on *Arabidopsis 35S:GUS-tRNA*$^{Met\text{-}dDT}$ (**A-D**) and *35S:GUS-tRNA*^{Gly} (**E-H**). (**A** and **E**)Bright field image by upright microscope. (**B** and **F**) Transmission image by confocal laser scanning microscopy. (**C** and **G**) Fluorescent image of aniline blue-stained sieve plates by confocal laser scanning microscopy. (**D** and **H**) (D) and (F) are overlay image of (B) and (C), (F) and (G) respectively. 20 μm parafin section of *Cuscuta* stem was stained by X-gluc for 24 hours and aniline blue for 45 minutes. Red arrows indicate GUS stained cells. Yellow arrows indicate aniline-blue-stained sieve plates. Scale bar: 20 μm.
