# Supplementary material for: Mobile Host mRNAs Are Translated to Protein in the Associated Parasitic Plant Cuscuta campestris
Source: Plants (Basel). 2021 Dec 28;11(1):93. doi: 10.3390/plants11010093 (PMC8747733; doi:10.3390/plants11010093)
Supplement: Supplementary file 1 [file plants-11-00093-s001.zip › plants-1490272-sm/Supp_Tables1-2_20211103.docx]

**Supplementary Table 1**. β-Glucosidase activities in host *Arabidopsis* plants. Activity per mg protein using 4-MUG as the substrate was measured in extracts from *Arabidopsis* leaves. 4-MUG is 4-methylumbelliferyl-β-D-glucuronide.

| **Name of lines** | **Mean**  (pmol MU/min/mg protein) | **Std Err Mean** |
| --- | --- | --- |
| *Col-0* | 0.00 | 0.05 |
| *35:GUS* | 47.12 | 3.53 |
| *35:ER-GUS* | 19.02 | 11.19 |
| *35S:GUS-tRNA^Met^* | 53.72 | 0.83 |
| *35S:GUS-tRNA^Met-dDT^* | 44.59 | 11.98 |
| *35S:ER-GUS-tRNA^Met^* | 52.56 | 1.15 |
| *35S:ER-GUS-tRNA^Met-dDT^* | 50.25 | 2.16 |

**Supplementary Table 2**. List of primers used in this study.

| Gene name | Direction Primer sequences | |
| --- | --- | --- |
| *GUS-RT-PCR* | Forward | CTGACCAAGGAACTCGACCC |
|  | Reverse | GCTCACCCACGAAGTTCTCA |
| *CcActin* | Forward | ACAGGGTGCTCCTCAGGGGC |
|  | Reverse | GCACAGGGTGCTCCTCAGGG |
| *GUS-qRT-PCR* | Forward | GAACTGGTGAACGACGGACT |
|  | Reverse | CCTCATGTTTGCCAAAGCCC |
| *AtMON1* | Forward | CTGTCTTCTCATCTCTTGTC |
|  | Reverse | TCTTGCAATATGGTTCCTG |
| *AtWRKT53* | Forward | GCAACGAAACAAGTCCAGAG |
|  | Reverse | GTCTTTACCATCATCAAGCCC |
| *ER-GUS* | Forward | ATGAAGGTACAGGAGGGTTTGTTCGTGGTGGCTGTTTTCTACCTTGCTTATACGCAGCTAGTCAAGGGGATGGTAGATCTGAGG |
| *ER-tRNA^Met^* | Reverse | TATCAGAGCCACCTTCGATC |
| *ER-tRNA^Met-dDT^* | Reverse | TATCAGAGCGGACCTGTGGGTTATG |
| *ER-tRNA^Gly^* | Reverse | TGCACCAGCCGGGAATTGAAC |
| *ER-tRNA^Ile^* | Reverse | GCTTCCGGCGGGGCTCGAAC |
